# A Study on the Efficient Preparation of α-Ketoglutarate with L-Glutamate Oxidase

**DOI:** 10.3390/molecules29081861

**Published:** 2024-04-19

**Authors:** Shuhui Niu, Fang Liu, Yaping Wang, Ben Rao, Yueying Wang

**Affiliations:** 1 National Biopesticide Engineering Technology Research Center, Hubei Biopesticide Engineering Research Center, Hubei Academy of Agricultural Sciences, Biopesticide Branch of Hubei Innovation Centre of Agricultural Science and Technology, Wuhan 430064, China; 13971485202@163.com (S.N.);; 2 State Key Laboratory of Biocatalysis and Enzyme, Engineering Hubei Collaborative Innovation Center for Green Transformation of Bio-Resources, Hubei Key Laboratory of Industrial Biotechnology, Biology Faculty, Hubei University, Wuhan 430062, China

**Keywords:** alpha-ketoglutaric acid (α-KG), L-glutamate oxidase, catalase, whole-cell catalysis

## Abstract

Alpha-ketoglutaric acid (α-KG), as an intermediate product of the tricarboxylic acid cycle, plays a crucial role in peptide and amino acid synthesis. In order to reduce costs and improve efficiency in the oxidative production of α-ketoglutaric acid, this study successfully synthesized and expressed L-glutamate oxidase (LGOX^Str^) from *Streptomyces viridosporus* R111 and catalase (KatG^Esc^) from *Escherichia coli* H736. Two immobilization methods and the conditions for one-step whole-cell catalysis of α-ketoglutaric acid were investigated. α-Ketoglutaric acid has broad applications in the pharmaceutical, food, and chemical industries. The specific research results are as follows: (1) By fusing the sfGFP tag, L-glutamate oxidase (LGOX^Str^ r) and catalase (KatG^Esc^) were successfully anchored to the outer membrane of *Escherichia coli* cells, achieving one-step whole-cell catalysis of α-ketoglutaric acid with a conversion efficiency of up to 75%. (2) Through the co-immobilization of LGOX^Str^ and KatG^Esc^, optimization of the preparation parameters of immobilized cells, and exploration of the immobilization method using *E.coli*@ZIF-8, immobilized cells with conversion rates of over 60% were obtained even after 10 cycles of reuse. Under the optimal conditions, the production rate of α-ketoglutaric acid reached 96.7% in a 12 h reaction, which is 1.1 times that of *E. coli*@SA and 1.29 times that of free cells.

## 1. Introduction

Alpha-ketoglutaric acid (α-KG), which has the molecular formula C_5_H_6_NO_5_ and is also known as α-oxo succinate or α-oxoglutarate, is a white powdery substance at room temperature. It easily dissolves in water and alcohol but is insoluble in ethers. Upon long-term storage, it turns pale grayish-yellow and is prone to hygroscopicity. α-KG, as an important intermediate product of the tricarboxylic acid cycle, plays a crucial role in the synthesis of amino acids and peptides. It also has broad applications in industry, pharmaceuticals, organic synthesis, and nutritional fortifiers [1,2,3,4].

L-glutamate oxidase (LGOX)-[EC 1.4.3.11] is a flavoprotein enzyme that oxidizes L-glutamate to α-ketoglutarate using flavin adenine dinucleotide (FAD) as a cofactor, without the need for additional FAD. LGOX possesses the advantages of mild reaction conditions and high catalytic efficiency and has extensive applications in the medicine, food, and chemical industries. Therefore, it has been a primary focus of research. LGOX can catalyze L-glutamate to produce α-ketoglutarate, ammonia, and hydrogen peroxide at room temperature, and it exhibits a specific catalytic effect on L-glutamate [5,6,7].

Catalase [EC 1.11.1.6] is an enzyme that decomposes H_2_O_2_ and can also react with various other substrates. With the continuous increase in the number of complete catalase sequences, researchers have divided them into three classes based on structure and function: typical monofunctional catalases (CATs), bifunctional catalase-peroxidases (KatGs), and manganese-containing catalases (MnCATs). CATs are present in aerobic respiration organisms but are usually not expressed in anaerobic bacteria. They typically exist as homotetramers, ranging from 200 to 340 kDa in size and containing four cofactors, with iron protoporphyrin IX being found in the active center (similarly to the prosthetic group in human hemoglobin). The uniqueness of KatGs lies in their higher activity than that of CATs under neutral conditions, as well as their superior affinity for substrate H_2_O_2_ and greater sensitivity to pH and temperature than that of typical catalases. For instance, horseradish peroxidase (HRP) is now widely used in Western blot experiments. MnCAT is a manganese-containing catalase in which manganese ions replace the iron–porphyrin structure [8,9,10].

The oxidative deamination process of L-glutamate (salt) to produce α-KG catalyzed by LGOX often accompanies the accumulation of H_2_O_2_. To eliminate the inhibitory impact of H_2_O_2_ on the production of α-KG, this project involved the use of LGOX coupled with catalase for the one-step preparation of α-KG. For this project, bifunctional catalase-peroxidase (KatG) was chosen to be coupled with LGOX for preparation. Some researchers have tried to co-express catalase and glutamate oxidase to reduce production costs, but due to different expression levels, adjusting promoters to regulate the expression levels of the two proteins requires significant gene engineering, and it is not easy to simultaneously grasp the expression levels of both enzymes [11,12].

In 1962, Shimomura et al. discovered a protein from Aequoria Victoria jellyfish that emits green fluorescence under ultraviolet light, which they named Green Fluorescent Protein (GFP), with a molecular weight of about 20 kDa. The fluorescence intensity of wild-type GFP decreases during fusion expression, and misfolding can easily occur during the expression process. However, modified GFP mutants have good fluorescence intensity and folding properties, making them suitable markers for fusion proteins. For example, the super-folded Green Fluorescent Protein (sfGFP) modified in this laboratory underwent six rounds of mutation based on GFP (frGFP), and its folding rate is not only 3.5 times that of frGFP, but it also has a higher expression level than that of frGFP. Compared with frGFP, sfGFP has greatly improved in terms of foldability, stability, and solubility [13,14,15,16].

Whole-cell immobilization has been widely used in industrial wastewater treatment, energy development, drug production, chemical production, etc. A suitable immobilization method has a crucial impact on enzymatic activity and yield, and the nature, type, production scale, reaction time, and reaction speed of the encapsulated object should be taken into consideration during selection. Currently commonly used immobilization methods include the following: embedding, adsorption, covalent bonding, and cross-linking. Sodium alginate (SA) is a common immobilization carrier that is widely used for cell immobilization due to its simple operation, mild conditions, and non-toxicity. Metal–Organic Frameworks (MOFs) are self-assembled metal–organic frameworks composed of metal ions and organic connectors. Different metal nodes and organic connectors can be combined to assemble many types of MOFs [17,18,19,20,21]. MOFs have the following advantages: (1) adjustable porosity; (2) easy material modification; (3) rich binding sites for specific targets; (4) good mechanical stability. MOFs are widely used as emerging immobilization carrier materials. ZIFs are some of the most stable materials in MOFs, and their stability in aqueous solutions is even more remarkable. ZIF-8, which is also known as zeolitic imidazolate framework-8, is a Metal–Organic Framework (MOF) material composed of zinc ions and imidazolate linkers. It has a three-dimensional porous structure with a large surface area, making it useful for applications such as gas storage, separation, catalysis, and drug delivery. ZIF-8’s unique properties stem from its crystalline structure, which allows for precise control over pore size and chemical functionality. This material has garnered significant attention in the fields of chemistry, materials science, and engineering due to its potential in various industrial and environmental applications [22].

As a kind of flavin enzyme, LGOX can specifically catalyze the deamination of L-glutamate to produce α-KG, NH_3_, and H_2_O_2_ using FAD as a cofactor. However, during whole-cell catalysis, the stability of free cells is poor, and the catalytic efficiency will significantly decrease when it is reused, which affects the industrial application of LGOX. Additional catalase is also needed in the reaction process to eliminate the effect of the byproduct H_2_O_2_ on α-KG. Therefore, using immobilization technology to improve the stability and reuse rate of whole-cell catalysis is a feasible solution. There are few reports about the immobilization of whole LGOX cells, so it is necessary to explore suitable and efficient immobilization methods. This study selected SA and ZIF-8 as immobilization carriers to co-immobilize L-glutamate oxidase and catalase, compared the effects of the two carriers through the production of α-KG, explored the preparation conditions of the two immobilization methods, optimized the impacts of single factors on both immobilization methods, and compared the transformation efficiency of the two immobilized cells. In the end, it was proven that ZIF-8 is a more advantageous carrier material than SA in this project. The catalytic performance and stability of *E. coli*@SA and *E. coli*@ZIF-8 compared to free cells were also explored.

## 2. Materials and Methods

### 2.1. Strains and Plasmids

The main reagents used in this experiment, PrimeStar Max, DNA Marker DL 2000, and DL 1 kb, were purchased from TaKaRa company (Dalian, China). Page RulerTM Prestained Protein Ladder and Ribo Lock RNase inhibitor were purchased from Thermofisher Scientific (Waltham, MA, USA). A Plasmid Miniprep Kit and Gel Extraction Kit were purchased from Omega Company (Biel/Bienne, Switzerland). Purple loading dye was purchased from Thermo Fisher Scientific. Alpha-ketoglutarate was purchased from Aladdin Reagent Co., Ltd. (Shanghai, China). L-glutamate and SA were purchased from Shanghai Macklin Biochemical Co., Ltd. (Shanghai, China). Glutaraldehyde was purchased from Sinopharm Group (Shanghai, China).

### 2.2. Construction of Recombinant Plasmids

The *E. coli* strain used for expression in this experiment was competent *E. coli* BL21(DE3) cells that were preserved in our laboratory. The *E. coli* cloning strains used in this experiment were competent *E. coli* DH5α and *E. coli* XL-GOLD cells that were also preserved in our laboratory. The plasmids and primers involved in this experiment are shown in Table 1 and Table 2, respectively.

In this study, the plasmids were constructed using the classical double digestion method. For single genes (LGOX^Str^/KatG^Esc^), double digestion (NotI/SacI) and T4 DNA ligation were used to clone them into the vector. Fusion proteins (sfGFP-LGOX^Str^/sfGFP-KatG^Esc^) were constructed using overlap extension PCR, followed by the same double digestion (*Not*I/*Sac*I) and T4 DNA ligation methods for cloning into the expression vector.

### 2.3. Cell Cultivation and Protein Expression

Competent cells of *E. coli* BL21(DE3) were transformed with the plasmid construct carrying the gene expressing LGOX^Str^ or KatG^Esc^. A freshly transformed colony was inoculated into LB media supplemented with 100 µg/mL kanamycin and incubated by shaking at 37 °C and 220 rpm for 16–18 h. On an ultra-clean worktable, the required seed solution was added to the TB medium in a certain proportion. When the OD_600_ was reached between 0.6 and 0.8, IPTG with a final concentration of 1 mM was added to induce the expression of the required recombinant protein, and then it was placed on a 220 rpm shaking table at 18 °C for 18 h or extended according to other conditions. The medium containing a large number of bacteria was introduced into the centrifuge and centrifuged for 10 min with a high-speed centrifuge at 6000× *g* rpm. After the supernatant waste liquid was dumped, ultra-pure water was added to re-suspend the cells, and then they were centrifuged again to collect the cells, which were placed at −80 °C for low-temperature preservation.

### 2.4. Enzyme Activity and Substrate Detection Methods

The enzyme reaction mixture was prepared according to the following system: 1.4 mL of 0.261 μg/mL N, N-dimethylaniline, 1 mL of 0.121 μg/mL 4-aminophenazone, 0.5 mL of 11 μg/mL L-glutamic acid, and 100 μL of 60 U/mL horseradish peroxidase. After mixing the above reagents, 100 μL of enzyme solution was added and allowed to react for 30 min; then, the absorbance at 550 nm was measured. The LGOX^Str^ activity was defined as the amount of enzyme required for the formation of 1 μmol of H_2_O_2_ in one minute under the most suitable catalytic reaction conditions of the enzyme [22].

Peroxidase can catalyze the oxidation of guaiacol by H_2_O_2_ to produce 4-methoxyphenol, and the characteristic absorption peak of its product is at 470 nm, which can be detected by the change in absorbance.

The content of α-ketoglutarate was determined with HPLC (Agilent HP 1100, Los Angeles, CA, USA). Separation column: Agilent C18 (Octadecylsilane-bonded silica gel, 5 μm, 4.6 mm × 250 mm); mobile phase: phosphate buffer (pH 2, 50 mM) containing 1% acetonitrile; flow rate: 0.5 mL/min; column temperature: 35 °C; injection volume: 10 μL. At around 9.26 min, the absorption peak of α-ketoglutarate could be detected at 245 nm. A concentration gradient was prepared for α-ketoglutarate standard samples as described above, with 3 replicates at each concentration, and the standard curve was plotted.

### 2.5. Preparation of Immobilized Cells

SA immobilization of whole cells: First, 0.5 g of LGOX^Str^ and KatG^Esc^ whole-cell mixture was weighed and mixed thoroughly with 50 mL of 2% SA solution, and an appropriate amount of the mixture was drawn with a 1 mL syringe. It was slowly dropped into a 1% CaCl_2_ solution to form small spherical particles. After standing for 1.5 h, the solution was filtered out, and the spheres were stored in a 0.9% NaCl solution at 4 °C.

ZIF-8 immobilization of whole cells: First, 0.5 g of LGOX^Str^ and KatG^Esc^ whole-cell mixture was weighed and mixed thoroughly with 10 mL of a 300 mM 2-methylimidazole precursor solution with a pH of 7. After 10 min, a final concentration of 40 mM glutaraldehyde (GA) solution was added, and stirring was continued for another 10 min. Finally, 10 mL of 100 mM zinc acetate solution was added, allowed to stand for 0.5 h, and then filtered and recovered for use.

### 2.6. Optimization of Single Factors for Immobilized Whole Cells

The single-factor optimization of SA-immobilized whole cells involved adjusting various parameters while keeping all other components constant. The parameters optimized were the SA concentration (1–6% gradient), cell concentration (5–40 g/L gradient), CaCl_2_ concentration (0.5–5% gradient), and immobilization time (various time gradients). Following each change, *E. coli*@SA was prepared according to the mentioned procedure and was reacted with the same substrate concentration. The α-KG content was then measured with the reaction gradient set in triplicate for each parameter. The group with the highest accumulated product quantity represented 100%, with all others being relative.

The single-factor optimization of ZIF-8-immobilized whole cells involved modifying parameters such as the concentrations of 2-methylimidazole (100–1000 mM gradient), zinc acetate (20–200 mM gradient), and glutaraldehyde (10–100 mM gradient) and the amount of encapsulation (5–40 g/L gradient). All other components are maintained constant. For each parameter, *E.coli*@ZIF-8 was prepared using the indicated method and was reacted with the same substrate concentration. The α-KG quantity was then determined, with the highest product accumulation set to 100% and all others as relative values. This process was conducted in triplicate for each parameter.

### 2.7. Optimization of the Conversion Conditions for the Catalytic Generation of α-KG

Substrate concentration optimization: To investigate the effects of different substrate concentrations on the production of α-KG, a transformation system was set up with 10 g/L of free cells and immobilized cells, an L-glutamate solution gradient concentration (20, 40, 60, 80, and 100 g/L), and a phosphate buffer with a pH of 7.0. The experiment was conducted at 30 °C on a shaker (220 rpm) for 12 h of transformation. The yield of α-KG was measured after the reaction.

Reaction temperature optimization: Free cells and immobilized cells were placed under different temperatures (20, 25, 30, 35, 40, 45, 50 °C) for experimentation; the transformation system was the same as that described in Section 2.6, with a substrate concentration of 40 g/L. The yield of α-KG was measured after the reaction.

Reaction pH optimization: Free cells and immobilized cells were placed in phosphate solutions with different pH values (5.0, 5.5, 6.0, 6.5, 7.0, 7.5, 8.0, 8.5, 9.0); the transformation system was the same as that described in Section 2.6, with a substrate concentration of 40 g/L. The yield of α-KG was measured after the reaction.

Repeated batch experiments: Free cells and immobilized cells were used separately for conversion experiments to catalyze the production of α-KG from L-glutamate. The transformation system was the same as that described in Section 2.6, with a substrate concentration of 40 g/L. After the reaction was completed, the immobilized cells and free cells were centrifuged and collected for subsequent batch experiments.

Storage performance experiments: Free cells and immobilized cells were stored at 4 °C. Every 24 h, specific amounts of immobilized cells and free cells were selected for conversion experiments, with the yield from the cells reacting with the substrate before storage being used as initial data. The residual activity of the cells within 14 days was observed.

Comparison of the thermal stability of free cells and immobilized cells: Free cells and immobilized cells were put into a water bath at 30–70 °C and arranged in a time gradient (0.5 h, 1 h, 1.5 h, 2 h, 2.5 h, 3 h, 3.5 h, 4 h). After cooling down, they were reacted with the same concentrations of substrates, with three parallel experiments being set up, after which the yield of α-KG was measured.

Comparison of conversion rates in the catalytic generation of α-KG: Under optimal conditions, free cells and two types of immobilized cells were separately reacted with 40 g/L of the substrate for 12 h. The yield of α-KG was measured according to the method described in Section 2.4, and the conversion rate was calculated.

## 3. Results and Discussion

### 3.1. Expression and Purification of Recombinant Proteins

**Figure 1:**
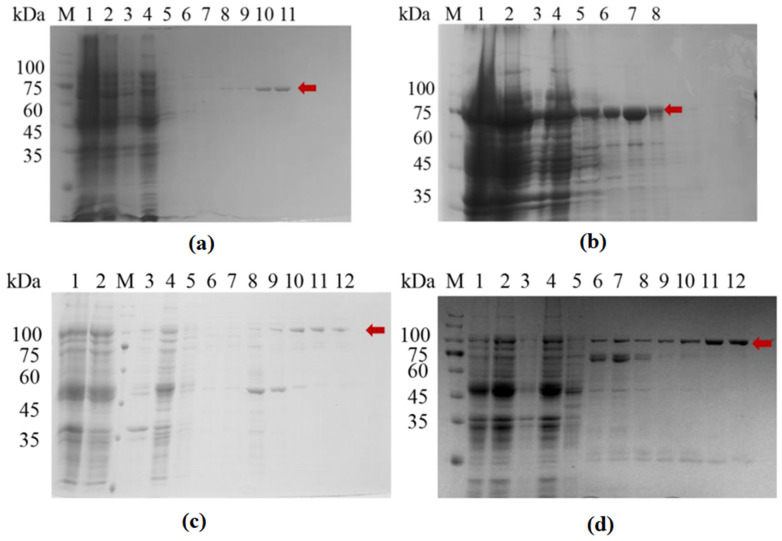
Protein expression and purification diagram. (**a**) LGOX^Str^; (**b**) KatG^Esc^; (**c**) sfGFP-LGOX^Str^; (**d**) sfGFP-KatG^Esc^; M: Protein MARKER; Lane 1: Whole cells; Lane 2: supernatant after cell lysis; Lane 3: resuspension of cell lysis pellet; Lane 4: column flow-through; Lanes 5–12: elution with 10 mM, 20 mM, 50 mM, 100 mM, 200 mM, 300 mM, and 400 mM imidazole solutions. The red arrow indicates the target protein.

The resuspended bacterial cells were lysed using a high-pressure homogenizer, followed by affinity purification using a Ni-NTA column. The results of SDS-PAGE analysis of the purified proteins are shown in Figure 1, and the detected bands are consistent with the expected sizes of the two proteins (SFGFP-LGOX^Str^: 95 kDa, sfGFP-KatG^Esc^: 103 kDa).

In the SDS-PAGE analysis, relatively pure target proteins could be obtained within the range of 100–400 mM for the imidazole elution solution. The components from the purer gradient of elution were collected for ultrafiltration and buffer exchange, followed by Western blot analysis.

### 3.2. Western Blot Identification of Recombinant Protein

**Figure 2:**
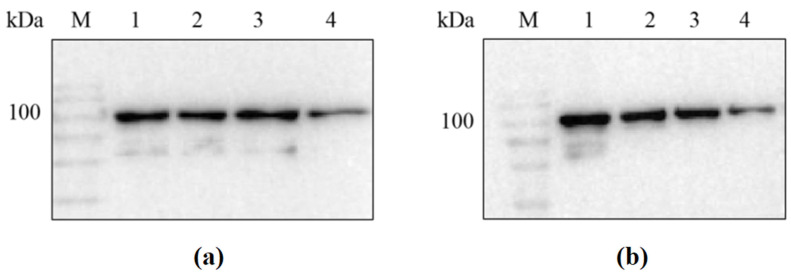
Western blot analysis of sfGFP-**LGOX^Str^** and sfGFP-**KatG^Esc^** proteins. (**a**) M: protein marker; lane 1: sfGFP-LGOX^Str^ washing membrane supernatant; lanes 2, 3: sfGFP-LGOX^Str^ purified protein; lane 4: sfGFP-LGOX^Str^ bacterial lysis precipitation resuspension; (**b**) M: protein marker; lane 1: sfGFP-KatG^Esc^ washing membrane supernatant; lanes 2, 3: sfGFP-KatG^Esc^ purified protein; lane 4: sfGFP-KatG^Esc^ bacterial lysis precipitation resuspension.

Since the 6× His tag was placed at the N-terminus of the recombinant protein during vector construction, the success in obtaining the target recombinant proteins sfGFP-LGOX^Str^ and sfGFP-KatG^Esc^ could be verified through the results of the Western blot (see Figure 2).

The results showed that the detected bands were consistent with the expected sizes of the two proteins (95 kDa and 103 kDa). This indicates that sfGFP-LGOX^Str^ and sfGFP- KatG^Esc^ had a soluble expression in *Escherichia coli* BL21(DE3), and the proteins were successfully anchored to the outer membrane under the action of the sfGFP tag, allowing for whole-cell catalysis.

### 3.3. Exploration of the Bacterial Biomass Proportion in the Alpha-Ketoglutarate Synthesis System

**Figure 3:**
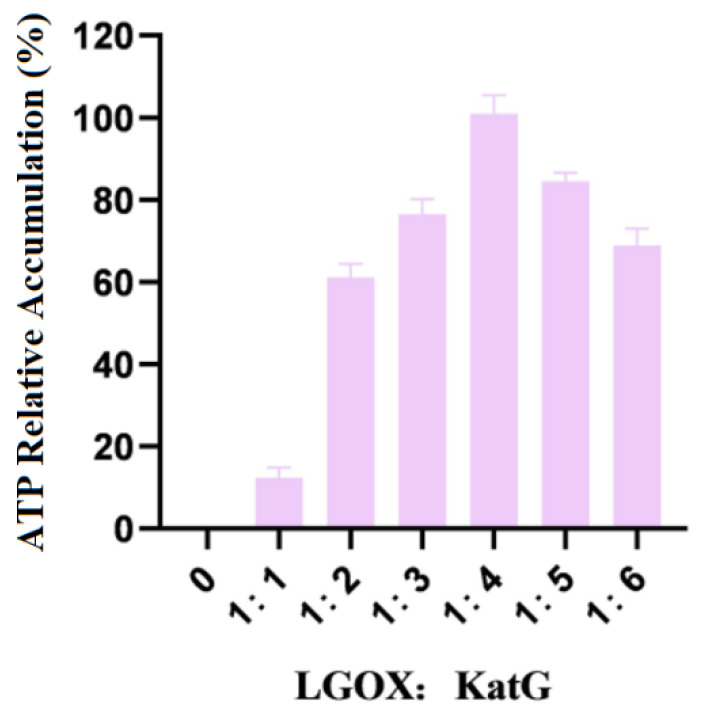
Ratios of the addition of LGOX and KatG to bacteria.

The mixture of whole LGOX^Str^ and KatG^Esc^ cells was weighed so that the wet weight ratios were 1:1, 1:2, 1:3, 1:4, 1:5, and 1:6, respectively, and they were reacted with 100 mmol/L L-glutamic acid. The amounts of products accumulated in the experimental group with the highest data were treated as 100%. The experimental results are shown in Figure 3.

As shown in the figure, when LGOX^Str^ was constant and KatG^Esc^ was variable in the system, the accumulation of the products no longer increased when the wet weight ratio of the two types of bacteria reached 1:4. Therefore, the optimal ratio of LGOX^Str^ to KatG^Esc^ in the synthetic system was 1:4. In this set of experiments, 100% corresponds to an α-KG yield of 0.11 g/L.

### 3.4. Analysis of Immobilized Cell Characterization Results

**Figure 4:**
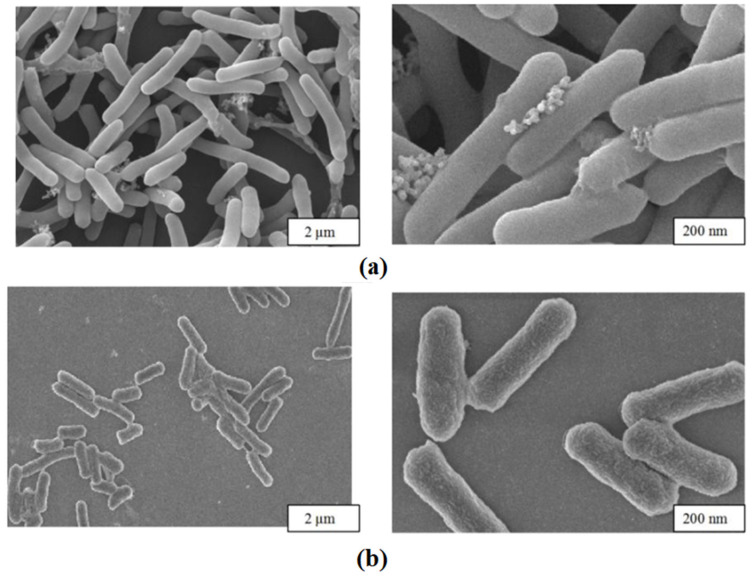
Scanning electron microscopy. (**a**) Immobilized cells; (**b**) free cells.

The morphology of immobilized cells and free cells was observed through Scanning Electron Microscopy (SEM), as shown in Figure 4.

The figure shows the appearance of *E. coli*@ZIF-8 and free cells under an electron microscope. Compared to free cells, deposits of ZIF-8 shells were observed on the surface and around the immobilized cells, and the cells were more tightly bound. At the same time, it could be observed that the integrity of the cells was good, reducing the loss of activity.

### 3.5. Single-Factor Experiment on the Two Immobilization Methods

#### 3.5.1. SA Whole-Cell Immobilization

**Figure 5:**
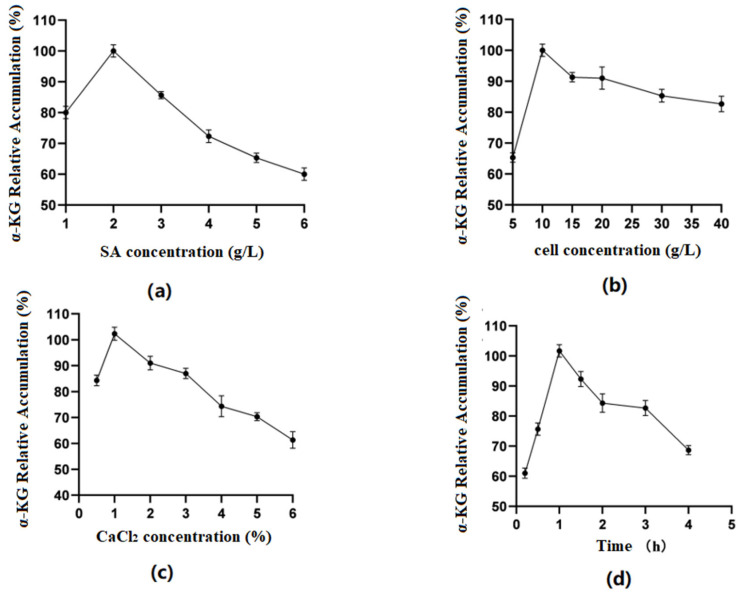
(**a**) Optimal sodium alginate concentration; (**b**) optimal cell concentration; (**c**) optimal CaCl_2_ concentration; (**d**) optimal fixation time.

Optimization of SA concentration (Figure 5a): The results indicated that when the SA concentration was between 1 and 6%, the amount of α-KG produced by the fixed whole cells reacting with the substrate increased first and then decreased as the SA concentration increased. At a concentration of 2%, the relative accumulation of the product reached its highest. The reason might have been that as the concentration of SA increased, the volume of the microspheres themselves gradually increased, the surface area of cells binding to the substrate decreased, and the catalytic efficiency correspondingly decreased. Therefore, in the experiment, the 2% SA concentration was chosen as the best.

Optimization of cell concentration (Figure 5b): As shown in the figures, when the cell concentration was 5–10 g/L, the enzyme activity of immobilized cells showed an upward trend. When it was within the range of 10 g/L–40 g/L, the enzyme activity tended to decrease. Excessive cell concentrations exceeded SA’s carrying capacity for cells, obscuring the active center of the enzyme and reducing the reaction activity. Therefore, this experiment used 10 g/L of cell volume.

Optimization of CaCl_2_ concentration (Figure 5c): It was found in the experiment that when the concentration of CaCl_2_ was 0.5%, microspheres could not be calcified well, but when the concentration was above 1%, the microspheres were too hard. Therefore, 1% was considered the ideal concentration of the CaCl_2_ solution.

Optimization of fixation time (Figure 5d): The results showed that when the time was 0.5–1 h, the accumulation of α-KG first increased and then decreased with time. The preliminary analysis showed that the longer the microgel particles calcified in the CaCl_2_ solution, the smaller the pore size of the carrier would be, making it difficult for the enzyme molecules to bind to the substrate. Therefore, 1 h was selected as the best fixation time.

#### 3.5.2. ZIF-8 Whole-Cell Immobilization

**Figure 6:**
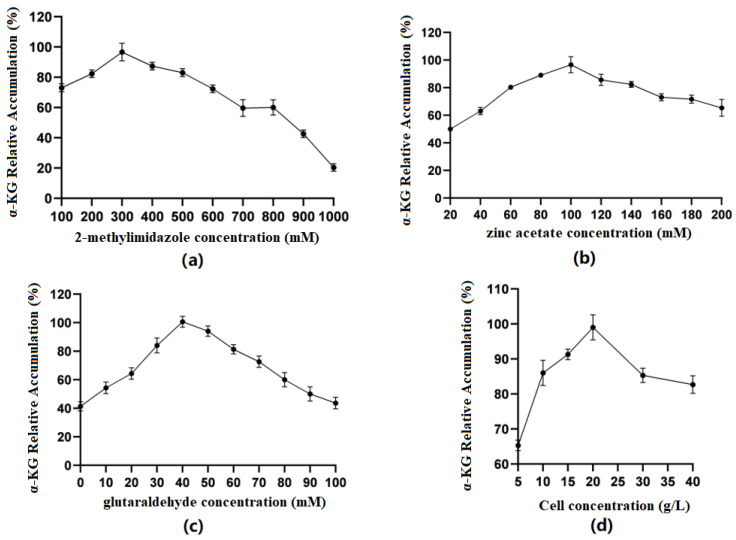
(**a**) Optimal concentration of 2-methylimidazole; (**b**) optimal concentration of zinc acetate; (**c**) optimal concentration of glutaraldehyde; (**d**) optimal cell concentration in the ZIF-8 synthesis system.

Optimization of 2-methylimidazole concentration (Figure 6a): The results showed that when the concentration of 2-methylimidazole gradually increased within a limited range, the catalytic efficiency of immobilized cells first increased and then decreased. When the concentration was 300 mM, the relative accumulation of the product was the highest. The preliminary speculation was that different concentrations of 2-methylimidazole could affect whether ZIF-8 was evenly coated on the surface of the cells.

Optimization of zinc acetate concentration (Figure 6b): The results showed that when the concentration of zinc acetate was between 20 and 100 mM, the production of α-KG gradually increased with the increase in concentration. When the concentration of zinc acetate reached 100 mM, the catalytic efficiency of immobilized cells was the best. Zn^2+^ coordinated with the imidazole group to form ZIF-8. When the concentration of 2-methylimidazole was constant, the concentration of Zn^2+^ could affect the formation of ZIF-8 and, thus, affect the cell activity.

Optimization of glutaraldehyde concentration (Figure 6c): The results showed that when the concentration of GA was 0–40 mM, the production of α-KG gradually increased with the increase in concentration. When the GA concentration reached 40 mM, the catalytic efficiency of immobilized cells was the best. GA, as a common crosslinking agent, had an aldehyde group that could react non-specifically with the amino group on the cell membrane, playing a role in structural fixation and increasing the cell stability. However, glutaraldehyde itself has a certain toxicity, and when its concentration is too high, it will cause some damage to cells.

Optimization of embedding amount (Figure 6d): The results showed that when the cell volume was between 5 and 20 g/L, the production of α-KG gradually increased with the increase in concentration. When the cell concentration reached 20 g/L, the catalytic efficiency of immobilized cells was the best. Then, there was a significant decline, which may have been because when the cell concentration was too high, the ZIF-8 coating on the cell surface was uneven, thus affecting the catalytic efficiency.

### 3.6. Optimization of Conversion Conditions for the Production of α-KG by Free and Immobilized Cells

**Figure 7:**
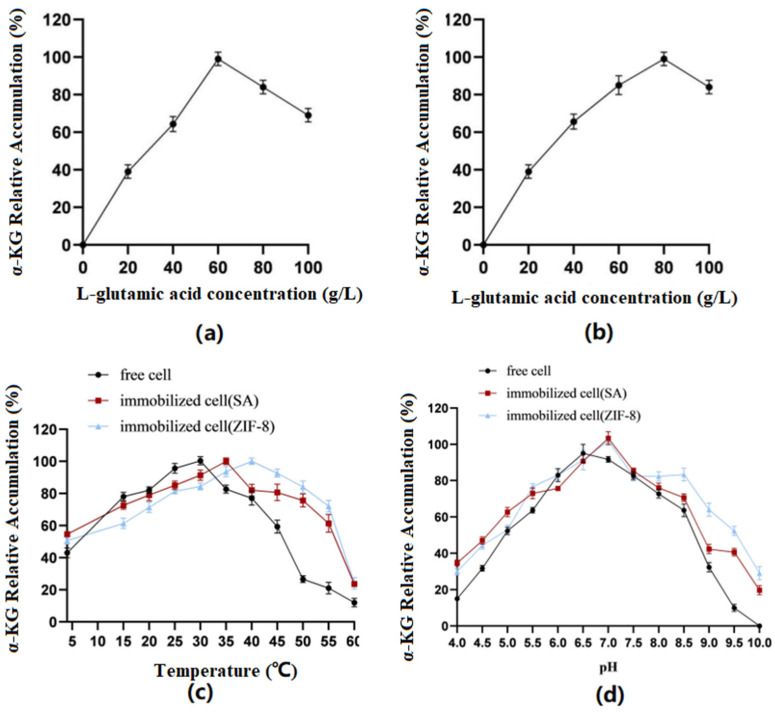
(**a**) SA: Conversion efficiency of immobilized cells for substrates of different concentrations. (**b**) ZIF-8: Conversion efficiency of immobilized cells for substrates of different concentrations. (**c**) Optimal temperatures of immobilized and free cells. (**d**) Optimal pH of immobilized and free cells.

Optimization of substrate concentration: The results (Figure 7) showed that the immobilized cells using SA as the carrier had the best catalytic effect when catalyzing the conversion of the substrate at a concentration of 60 g/L. When the concentration was greater than 60 g/L, it was not conducive to product accumulation. However, when ZIF-8 was used as the carrier, the substrate concentration could be increased to 80 g/L.

Optimization of reaction temperature: The results showed that the optimal temperature for free cells was 30 °C, the optimal temperature for SA-immobilized cells was 35 °C, and the optimal temperature for ZIF-8-immobilized cells was 40 °C.

Optimization of reaction pH: The results showed that the optimal pH for free cells was 6.5, while the optimal pH for both immobilized cell types was 7.0. Furthermore, the immobilized cells exhibited better tolerance under alkaline conditions.

### 3.7. Repeat Batch Experiments on Free Cells and Immobilized Cells

**Figure 8:**
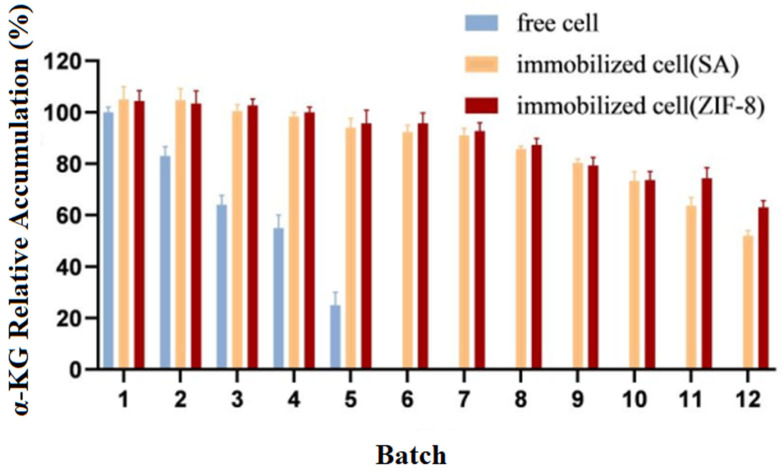
Operational stability of immobilized and free cells.

Experiments were carried out to catalyze the production of α-KG from L-glutamate separately with immobilized cells and free cells. Upon the completion of the reaction, the cells were collected via centrifugation and then washed three times with phosphate buffer before proceeding to the next batch transformation experiment. The relative accumulation of the products from different batches (1–12) was compared, with the highest value in the experiment being set to 100%. The experimental results are shown in Figure 8.

The results showed that compared to the free cells, the reusability and catalytic efficiency of the immobilized cells were significantly improved. After being used five times, the free cells almost lost their activity, while both types of immobilized cells could be reused for up to 12 batches, with *E. coli*@ZIF-8 performing even better.

### 3.8. The Storage Stability of Free Cells and Immobilized Cells

The free cells and immobilized cells were stored at 4 °C, and the same amounts of immobilized cells and free cells were taken every 24 h to conduct transformation experiments. The accumulation of the products was measured for 14 days while considering the amount of product before storage of the cells as 100%.

**Figure 9:**
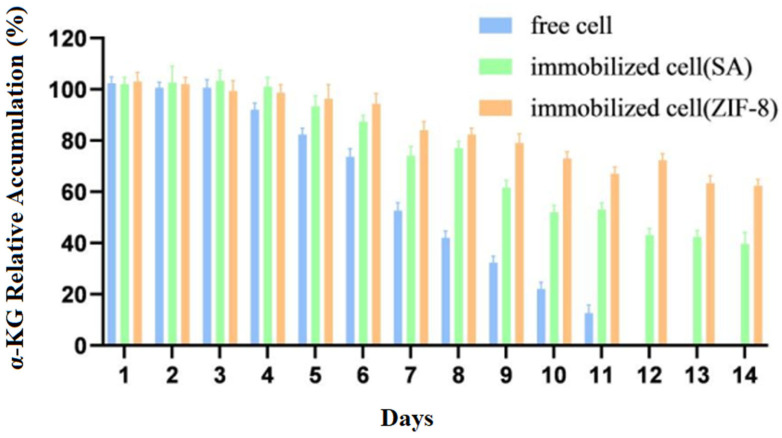
Storage stability of immobilized and free cells.

Compared to those of free cells, Figure 9 showed that the stability and catalytic efficiency of the immobilized cells were significantly improved. The accumulation of the products was measured for 14 days while considering the amount of product before the storage of the cells as 100%.

### 3.9. Comparison of Temperature Stability between Free Cells and Immobilized Cells

**Figure 10:**
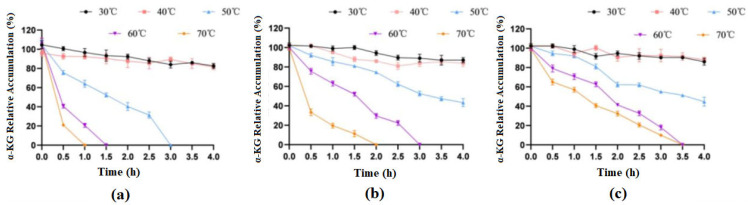
Effects of different temperatures on the catalytic activities of free and immobilized cells. (**a**) free cells; (**b**) cells immobilized on SA; (**c**) cells immobilized on ZIF-8.

After treating the immobilized cells and free cells at different temperatures for different durations, the whole cells preserved at 4 °C were used as a control group for examination, and the highest product accumulation values from the experiment were treated as 100%. The experimental results are shown in Figure 10.

The results showed that when treated at 30 °C and 40 °C for 4 h, the enzymatic activity of both free cells and immobilized cells basically did not change. When free cells were treated at 50 °C for 3 h, they basically lost their activity, but at this time, the catalytic efficiency of both types of immobilized cells could still stay around 50%. When treated at 70 °C for 1 h, free cells almost lost their catalytic activity, while SA-immobilized cells still had 19% activity, and ZIF-8-immobilized cells had 56% activity. In general, immobilized cells had significantly improved thermal stability compared to that of free cells, and ZIF-8 carriers were more advantageous.

### 3.10. Comparison of the Conversion Rate in the Catalytic Generation of α-KG

Under optimal conditions, free cells and two types of immobilized cells were each reacted with a substrate of 40 g/L for 12 h; the yield of α-KG was measured, and the conversion rate was calculated.

**Figure 11:**
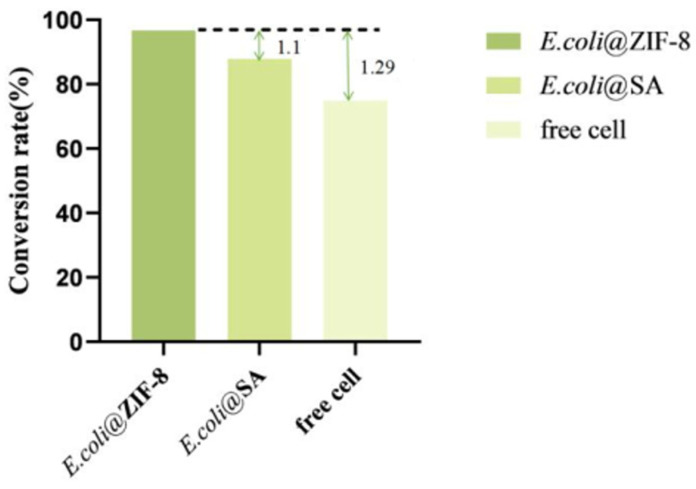
Comparison of the α-KG conversion rates.

According to the results (Figure 11), under optimal conditions, *E. coli*@ZIF-8 could catalyze the production of 38.4 ± 1.2 g/L of α-KG using L-glutamate (40 g/L) as the substrate, *E. coli*@SA could catalyze the production of 34.9 ± 2.3 g/L of α-KG, and free cells could catalyze the production of 29.74 ± 1.5 g/L of α-KG. The conversion rate of *E. coli*@ZIF-8 was 96.7%, which was 1.1 times that of *E. coli*@SA and 1.29 times that of free cells. In conclusion, by co-immobilizing LGOX^Str^ and KatG^Esc^ to achieve a one-step catalytic preparation of α-KG from L-glutamate, the yield of α-KG can be effectively increased, achieving efficient production.

## 4. Conclusions

The superfolder green fluorescent protein (sfGFP) modified in this experiment is based on the wild-type GFP (frGFP) and underwent six rounds of mutation. The correctly folded protein not only has a folding rate that is 3.5 times higher than that of frGFP but also has a higher expression level. Compared to frGFP, sfGFP experienced significant improvements in foldability, stability, and solubility. In addition, sfGFP has excellent temperature stability, maintaining some activity even after 30 min of treatment at 70 °C, providing a shortcut for purifying thermostable proteins.

The oxidative deamination process of LGOX^Str^ catalyzing L-glutamate (salt) to produce α-KG often accompanies the accumulation of H_2_O_2_, which limits the reaction rate and is not conducive to product accumulation. To eliminate the inhibitory effect of H_2_O_2_ on the production of α-KG and promote the catalytic efficiency of the reaction, it is necessary to add catalase to the reaction solution. This study simultaneously immobilizes the catalase (KatG) and L-glutamate oxidase (LGOX^Str^) on the cell surface using an sfGFP label. By inducing and adjusting the activity levels of KatG and LGOX^Str^, this method is simpler and can significantly guarantee the forward reaction, shortening the reaction time and saving costs.

This study cleverly solves the synergy problem between KatG and LGOX^Str^ through the sfGFP fusion and immobilization strategy. On one hand, the fusion of sfGFP mediates the anchoring of KatG and LGOX^Str^ on the cell surface, eliminating the need for cell lysis and simplifying the experimental process. On the other hand, the enzymatic activity levels of KatG and LGOX^Str^ in large-scale fermentation are unstable, and the relative enzymatic activity levels of the two enzymes directly determine the overall reaction rate and substrate conversion. The surface display enables the direct measurement of enzymatic activity levels, determining the addition ratio of the two strains in immobilized cells based on the relative enzymatic activities, which is convenient and efficient.

Meanwhile, the sfGFP fusion expression strategy can also allow the toxicity of the target protein to the host cells to be avoided, allowing the product to accumulate continuously without degradation. By utilizing the characteristics of sfGFP, which promotes protein solubility, this study successfully anchors the target protein on the outer membrane of *E. coli* cells, and subsequent experiments can use whole-cell catalysis without the need for cell lysis or purification. This lowers the production cost of α-KG, making it suitable for large-scale industrial production.

## Figures and Tables

**Table 1:** The plasmids used in the experiment.

Plasmid Name	Characteristic
pET28a-LGOXStr	T7 Promoter, Kanar, N-His
pET28a-KatGEsc	T7 Promoter, Kanar, N-His
pET28a-sfGFP-LGOXStr	T7 Promoter, Kanar, N-His
pET28a-sfGFP-KatGEsc	T7 Promoter, Kanar, N-His

**Table 2:** Primers in this study.

Primers	Sequence (5′-3′)
T7-F	TAATACGACTCACTATAGC
T7-R	GCTAGTTATTGCTCAGCGG
28a-F 28a-R	GGTGGTGCTCGAGCAGATCCTCACGATACAGCGG GGTGGTGCTCGAGCAGATCCTCACGATACAGCGG
sfGFP-F	ATGGTGAGCAAGGGCGAGGAGCTGT
sfGFP-R	CATTGGACCCTGGAACAAAACCTCCAATTTATACAGTTCA
LGOX^Str^-F	TGTTCCAGGGTCCAATGGCTTTGCCAGCTCCAGCTGCTTCTGG
LGOX^Str^-R	ATGATGATGATGATGAGCAGTGTGGATTTCCAAAG
KatG^Esc^-F	TTGTTCCAGGGTCCAATGCCATGAGCACCAGCGATGA
KatG^Esc^-R	ATGATGATGATGATGCTCGAGTTAATGATGATGATGAT

## Data Availability

Data are contained within the article.

## Abbreviations

LGOX^Str^	L-glutamate oxidase from *Streptomyces viridosporus* R111
KatG^Esc^	catalase from *Escherichia coli* H736
SA	sodium alginate
ZIF-8	zeolitic imidazolate framework-8
